# Understanding Vulnerability During Preventive Child Health Examinations: Insights from Danish General Practitioners

**DOI:** 10.3390/children13020221

**Published:** 2026-02-04

**Authors:** Sarah Kornum Melgaard, Lotte Lykke Larsen, Janus Laust Thomsen, Camilla Hoffmann Merrild

**Affiliations:** Center for General Practice, Aalborg University, 9260 Gistrup, Denmark

**Keywords:** child vulnerability, preventive child health examination, general practice

## Abstract

**Highlights:**

**What are the main findings?**

- General practitioners associate child vulnerability with multiple factors but findings during preventive child health examinations are mainly somatic.
- To assess child vulnerability, knowledge of the family is essential, which necessitates better collaboration with other sectors.

**What are the implications of the main findings?**

- Child vulnerability in general practice is a complex term.
- The preventive child health examination represents one of several opportunities to assess child vulnerability.

**Abstract:**

Background/Objectives: Child vulnerability is a predictor for potentially adverse challenges for the child and family, but the term is used inconsistently across settings. Danish general practitioners (GPs) are centrally positioned as the front line of the health care system. Thus, the aim of this study is to explore Danish GPs’ perspectives and assessment of child vulnerability, using an exploratory, sequential mixed-methods approach. Methods: Eleven GPs were interviewed, focusing on perceptions and management of child vulnerability in the context of preventive child health examinations (PCHEs). Interviews were analyzed in two stages. From the first deductive analysis, a quantitative data recording chart was developed. This was distributed to 10 general practices, to collect GPs’ perceptions and management of child vulnerability, and 197 recordings were completed. Secondly, to develop themes independently of the deductive coding, data was analyzed inductively, creating an in-depth understanding of GPs’ perspectives. This resulted in four themes. Results: GPs associated the concept of child vulnerability with a multitude of social, somatic, and psychological factors. To recognize child vulnerability, GPs found time and scope during PCHEs limited and knowledge of the family essential. Collaboration with social services was perceived as insufficient. The most frequent finding during PCHEs was related to somatic challenges (60%) and follow-up consultation was the most frequent response (64%). Conclusions: GPs considered child vulnerability a complex term. Assessment of child vulnerability was strongly related to knowledge of the family, and usually based on several consultations, which underscores that PCHEs represent only one of several contexts where concerns are assessed and addressed.

## 1. Introduction

Child vulnerability is a key concept in child health and welfare research, often linked to risks such as maltreatment, domestic violence, or later-life health and social difficulties [1,2,3,4]. Yet, the term is used inconsistently across disciplines and professional settings, from being described as a basic characteristic of the human condition to susceptibility to harm or as social determinants of health [5,6,7]. Especially in the health and social care sector, vulnerability has been operationalized as inherent or pre-described risks [8], related to specific factors such as socioeconomic or parental factors, or health determinants related to health status or access to care [2,5,9,10]. However, recent calls for consideration in the use of the term have shed light on the potential challenges in labeling someone “vulnerable” or pre-describing risks to specific groups in a health and social care setting [5,8,11]. Therefore, it has been argued that to better understand child vulnerability and its implications, it is essential to understand different health and social care professionals’ perspectives on vulnerability.

However, identifying vulnerable children who require additional support remains a challenge across health and social care systems, and it has been shown that assessing child vulnerability poses a particular challenge within the healthcare sector, where the social and health-related dimensions of child vulnerability are intertwined [2,12,13,14,15]. Although several models exist to evaluate vulnerability in a health care setting [16], recent research also highlights the relational nature of vulnerability, showing how interactions between health care providers and families shape professional perceptions of which children are considered vulnerable [12,17,18].

In a Danish context, general practice serves as the front line of the health care system. Access to health care is offered free of charge, and almost all Danish citizens are registered with a general practitioner. Thus, GPs play a vital role in supporting and ensuring the health and well-being of children and families, through daily consultations on both acute and preventive matters [19,20,21,22]. All Danish children are offered seven preventive child health examinations (PCHEs) by their GP [23], and more than 90% of children attend their first three PCHEs [19]. Therefore, PCHEs create important opportunities for GPs to assess child vulnerability [21,24]. Recent Danish studies also indicate that an increased psychosocial focus during PCHEs is welcomed by both GPs and parents [25,26]. However, little is known about how GPs perceive and assess child vulnerability during PCHEs. The aim of this study is therefore to fill this knowledge gap by exploring how Danish general practitioners perceive and evaluate child vulnerability during PCHE.

## 2. Materials and Methods

We employed an exploratory sequential mixed-methods design [27,28] to firstly understand perceptions and assessment of child vulnerability during PCHEs in depth and secondly investigate if findings in a broader population could support and widen our understanding of the qualitative findings. Qualitative data collection was therefore consecutively combined with the development and collection of quantitative data: first, qualitative data from semi-structured interviews with general practitioners was collected. Then deductive analysis of interviews informed the development of a data recording chart which allowed quantitative data collection in a broader GP population. Finally, data from the data recording chart were integrated with results from a second inductive analysis of the interviews, conducted to ensure an in-depth understanding of GPs’ perspectives independently from the deductive coding.

### 2.1. Interviews

Using snowball sampling, participants were recruited through professional networks in the general practice environment. We focused on GPs with a special interest in child health. We carried out 11 semi-structured interviews with GPs from nine different general practices in Aalborg municipality in the North Denmark Region over a period of 4 months, until data saturation was reached [29] (see Table 1 for characteristics of GP participants). The interviews focused on potential indicators of vulnerability in a child and/or family, possible findings during preventive child health examinations indicating vulnerability, and possible responses to findings or assessed vulnerabilities. GPs may recommend or refer the family to the municipality’s support services called ‘municipal health services’ [22,30]. Here, appropriate social and psychological support is ensured. Responses therefore included collaboration with social services, use of municipal health services and experiences or challenges with any cross-sectoral collaboration, e.g., with social services, private specialists or hospitals. Participants were encouraged to use examples to describe experiences or challenges, which could further illuminate the concept of child vulnerability in a concrete and practical way. Written and oral consent was obtained from all participants.

The interviews were carried out by the second author, who was not acquainted with any of the participants beforehand. Four interviews were carried out online due to COVID-19 restrictions and seven were carried out in person in each participant’s general practice. All were audio-recorded and lasted between 24 and 46 min. Subsequently, the interviews were transcribed verbatim by the second author.

### 2.2. Data Analysis—Interviews

**Figure 1:**
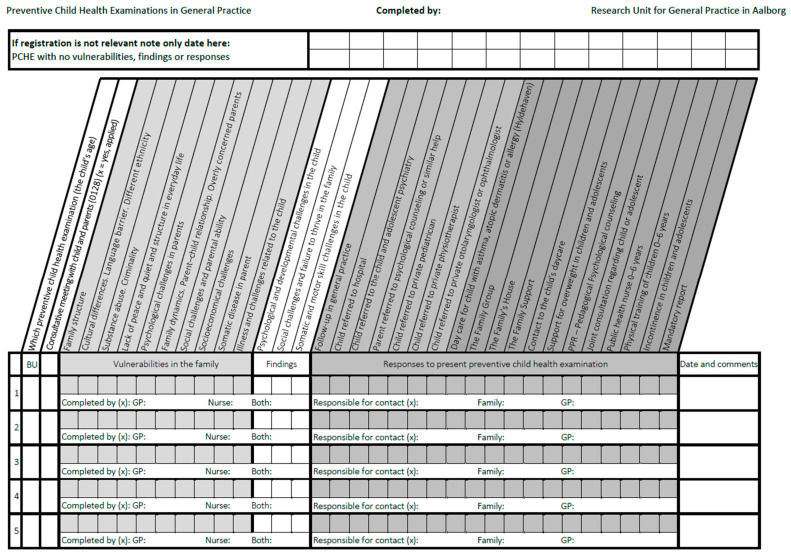
Data collection chart with topics of vulnerabilities, findings and responses used for quantitative data collection.

The data was analyzed in two stages. In the first stage, a deductive thematic analysis was carried out [31] with the purpose of developing the data recording chart. We identified 47 codes associated with vulnerabilities, 27 codes associated with findings during consultations and 20 codes associated with responses to the findings. Codes were grouped into themes and merged into topics by the second and last authors (Appendix A, Table A1, Table A2 and Table A3). Based on the themes developed and the municipal health services which were available for GPs online [32], the data recording chart was developed (Figure 1). The data recording chart contained 10 topics of vulnerability, 3 topics describing findings and 19 topics of responses. Topics of vulnerabilities and findings related to perceptions of child vulnerability, and interview respondents related the term “family” to immediate family (parents or caregiver), which was used as definition of the term in the subsequent data collection and analysis. Responses were organized as either municipal health service (12 topics) or non-municipal responses, e.g., private practicing specialists, hospitals or psychologists (7 topics) (Figure 1). To develop the data recording chart, we used the APO-structure method [33] for quality assessment in general practice, because it is a pragmatic concept and very simple to use and has been widely used to assess quality in treatment in general practice in Denmark in prior studies.

**Figure 2:**
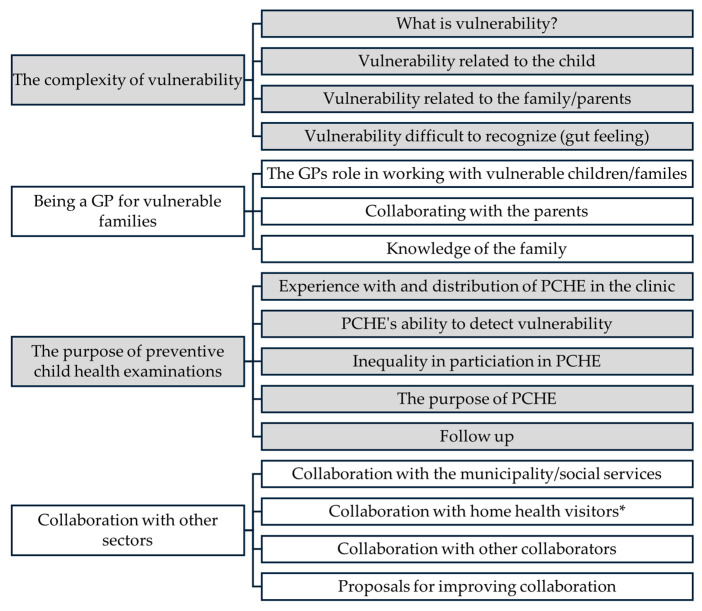
Codes and themes from second round of analysis of interviews: GPs’ perspectives on child vulnerability. * Nurse with a specialization in child health, five visits in the home in the first 10 months after birth, several visits to the child’s school with functional examinations during school age [35].

In the second stage of analysis, the data was re-read and re-coded by the first author. An inductive thematic analysis was carried out [31], to explore further perspectives or challenges experienced by GPs when providing care to vulnerable children during PCHEs. Themes were created manually while analyzing transcripts, and adjustments and additions were made iteratively during the process of analyzing all interviews [34]. Sixteen codes were created and merged into four themes under thorough discussions with the fourth author, resulting in no changes to codes or themes. In Figure 2, the coding and themes are displayed.

The double analysis of the interviews provided knowledge in two steps; initially it provided knowledge of relevant categories when systematically examining GP perspectives and reactions to child vulnerability in a broader population. Subsequently, it created in-depth knowledge of GPs’ perspectives on what constituted vulnerability and how to provide proper care and support for children living in vulnerable situations.

### 2.3. Data Recording Chart—Data Collection and Analysis

The data recording chart was pilot-tested using expert assessment in one general practice with seven GPs. Data was collected during two periods: in 2022 and 2024/2025. Each general practice applied the data recording chart to all PCHEs, to record vulnerabilities, findings or responses for each child. Examinations with no vulnerability, finding or response only required the examiner to note the date.

In the first period (2022), purposive sampling of clinics was used, restricted to practices in Aalborg municipality. No GPs from other municipalities were recruited due to local variations in municipal health services. Comparison of the latest accessible numbers of vulnerable children aged 0–17 years from the Danish registers showed the proportion of vulnerable children in Aalborg municipality as equal to the national proportion, although local variance in distribution could occur [36]. General practices from interviews were invited, and broad digital and physical networks of practices in Aalborg municipality were used to recruit other practices from the municipality. Three interviewed practices agreed to participate, two of which were located in urban areas and employed three GPs each, and one practice located in a suburban area, employing seven GPs. Before initiation of data collection, the second author visited the general practice, to inform the practice of the purpose of the project and explain the procedure for using the data recording chart. A total of 100 records were completed.

In the second period (2024/2025), one section in the data recording chart was modified before data collection, as municipal health services provided by Aalborg municipality had changed. Five changes were made to the topics of municipal health service responses. Participants were recruited using area-based, purposive sampling. All 47 general practices in Aalborg municipality were invited to participate. After invitation and two reminders, seven practices agreed to participate. Four were in urban areas, one in a suburban area and two in rural areas. The practices had between two and seven practicing GPs. The primary reasons not to participate were lack of resources or major organizational changes. During 2024/2025, 97 records were completed, making the total number of records 197.

Twenty-one records with no noted vulnerabilities or findings were excluded from the total number of recordings. Five records were excluded due to uncertainty regarding the response time, as this was not recorded in connection with PCHE. In total, 171 records were included in the study.

Data was summarized and analyzed descriptively by the first and second author using Excel. Responses were categorized as municipal health service or non-municipal, based on the structure of the data recording chart. The *p*-value for comparison of differences in distribution of pooled vulnerabilities and/or findings in total responses compared to “follow-up in general practice” was calculated by Wilcoxon rank-sum test using Stata 18, as normal distribution could not be assumed.

## 3. Results

### 3.1. Qualitative Data: Interviews

In the following, we present the four themes developed from the inductive analysis of the interviews:

Theme 1: The complexity of vulnerability

The concept of child vulnerability was the overarching focus of the project and therefore discussed in all interviews. GPs perceived child vulnerability as presenting in multiple potential forms, and related to social, somatic and psychological factors in the child or family, which were illuminated during the first deductive analysis. However, many respondents discussed their uncertainty about how to understand vulnerability, and considered the term difficult to grasp, with no single certain indicator:

*R1: “It […] is difficult to give a clear answer, just like that, to [whether there is a difference between findings in a vulnerable and non-vulnerable family], because there are certainly also resourceful families, who, especially with their first born child, are very, how to put it, on edge about even the smallest things, right […]. But [my] gut feeling is of course, that if you come across as, or if you have fewer resources, then it could probably also be harder to raise a child, and then something like… well, caregiving and all that can obviously fail now and then, right”.*

The appearance of and interaction in the family was considered significant, and some deemed this a primary factor in their considerations regarding what constitutes child vulnerability. In particular, psychiatric disease in a parent increased the GPs’ attention. However, respondents were aware of treading carefully when evaluating family relations, as this could sometimes also just be a product of societal norms.

Many respondents also underlined the difficulties of recognizing child vulnerability in general practice:

*R2 It’s like only looking at a landscape through a keyhole, when you are positioned in general practice, unless we know the family fairly well. And we don’t always do that. There’s this narrative, that […] the general practitioner knows the families, but if we reflect on our own role, and really look into that, I am actually not quite sure. And sometimes, I think it can get a bit in the way. ‘Well, that’s just that family, they’re like this or that’. But they might have gone through a divorce since last year, right, they might have gone through [a lot of things], they might have lost grandparents who were an important source of stability in the family. […] So, it’s definitely difficult to look into a child’s life through a single consultation… So, I find that challenging.*

GPs found somatic challenges easier to recognize, compared to psychological or social challenges. Many mentioned how known challenges related to the child or challenges detected in the physical examination could be an indicator of child vulnerability. However, some assigned this minor value as they would evaluate such findings in relation to knowledge of parents’ or caretakers’ resources.

Theme 2: Being a general practitioner for vulnerable families

As mentioned above, knowledge of the family was essential for many GPs when assessing vulnerable children and families. Many pointed to the difficulties in gathering this information during the regular PCHEs. Knowing any recent changes in the social or physical environment of the family was mentioned as highly relevant, but sometimes this background information was absent. Often, however, GPs encountered the mother during pregnancy. If this raised concerns, it could prompt the GP to initiate help or support early.

Respondents pointed out how the role of the GP was dependent on the collaboration and relationship with the parents. Some GPs considered their role during PCHEs to improve parenting skills and “raise” the parents, but many found it difficult to provide support to parents when concerns were less somatic. For instance, they often had the role of supporting and caring for frustrated parents, who sought help when confronted with social services. Here, the GPs felt they lacked opportunities to respond or act, and some were unsure whether this fell within their area of expertise:

*R8: “I have an example of a mother, a new mother, who came in and was known to have [a mental illness], and you could say there were major challenges in terms of […] supporting the mother in her contact with the infant, and supporting her in accepting help from the municipality and the family group [sic: support group in the municipality’s social services], and supporting her in how to handle having an infant, which of course put extra pressure on her. […] The advantage is that I […] know that I am available and a contact person going forward, but since many of these issues aren’t really about illness, it’s not like I, beyond being able to support and guide, have much more to offer. But I can encourage, uh… and notify the municipality, and maybe send a mandatory report, and things like that. Fortunately, she had a course of care where she was also in contact with a psychiatrist and the family group, and I think the family group in particular can be really helpful.”*

Many did, however, consider the support they could provide for the parents a key element in their role as a GP for vulnerable families.

Theme 3: The purpose of preventive child health examinations

The purpose of the preventive child health examination (PCHE) was debated through the interviews. Many respondents mentioned somatic assessment, e.g., height or weight, as their primary focus during PCHEs. Some worried that evaluating psychological or social challenges could jeopardize both the time frame and purpose of PCHEs. Others, however, found these parameters intertwined with the somatic assessment, or equally relevant:

*R8: “Of course, I have the things I think I need to look for during preventive child health examinations, but it’s also a free space for parents to share [what they’ve been thinking about], if there’s something we should talk about in relation to that. It’s a place where you can pose an open question: ‘[…] is there anything else you’ve been thinking about?’—which we can then address.”*

Many respondents mentioned their difficulties in recognizing vulnerabilities during the limited time frame of PCHEs. This was particularly the case when not acquainted with the family. Many applied follow-up consultations when struggling with something or suspecting something during the consultation:

*R6: “It’s about getting that suspicion regarding the things we examine during the preventive child health examination, and then being able to see them again to confirm the impression you had, when you think… there’s something here which isn’t quite right with the eye contact, or the child is crying in a strange way, or not gaining weight as expected, and the mother might seem, well, disengaged, flat. And so being able to see them again, to follow the progress, that, I think, actually provides a lot of information.”*

Respondents also described how PCHEs were partly delegated to other personnel, e.g., practice nurses. Some pointed out that this could compromise continuation and knowledge with the family, especially among vulnerable families, and therefore this delegation required thorough communication between practice nurses and GPs.

Theme 4: Collaboration outside general practice

None of the attending GPs received feedback from the municipalities’ social services after referral or report. Feedback on initiatives or interventions for the family was given by the parents, and only if the GP arranged follow-up consultations. Although some felt uncertain regarding the necessary amount of knowledge sharing with social services due to a possible inherent increase in workload, many emphasized a need for improved communication frequency and knowledge sharing with social services:

*R11: “I think it would be better if… this whole thing about if something is initiated within the municipality, then I would like to be more involved in it, or at least kept informed on an ongoing basis. Just to make sure, okay, this is under control, right? And then I can pick up the ball if the initiatives are terminated or something like that, right? That’s something I would really like.”*

Correspondence with the home health visitors (specialist nurse, part of municipal health services) was considered efficient by many respondents, although not regularly experienced. Many shared ideas on improving collaboration with social services, inspired by communication with home health visitors. Others underlined the fundamental challenges in collaboration centered around vulnerable children, e.g., mandatory reports:

*R2: “Well, it is very odd, the mandated reporting system […]. That’s the option we’ve agreed on. We’ve agreed that this is the path we use, even for small things, there’s been an attempt to normalize the mandatory report so it’s not just when you think mom and dad are hitting the child, but also when there are suspicions, even mild suspicions, about problems that need to be solved within the child and adolescent psychiatry or by the municipality who conduct the child welfare assessment. But most often, nothing happens. […] We’re supposed to make mandatory reports even for minor concerns, but it doesn’t work. Because the message doesn’t reach the right place, and we don’t know each other well enough.”*

Compared to the municipalities, the collaboration with the hospitals and other non-municipal health care services was efficient. Discharge summaries and status summaries were mentioned as essential instruments in facilitating knowledge sharing, thereby improving the GPs’ position in subsequent consultations with the patient.

### 3.2. Quantitative Data: Recordings During PCHEs

Turning towards the quantitative data on child vulnerabilities, the most frequently recorded vulnerabilities during PCHE were “illness + challenges related to the child” (33%), “psychological challenges in parents” (18%) and “family structure” (17%) (Table 2). In total, 40% of children displayed one vulnerability, and 3% displayed 5–7 vulnerabilities. In 35%, no vulnerabilities were assessed (Table 2).

The most frequently detected finding during PCHEs was “somatic + motor skills challenges” (60%) (Table 3). Most children had one detected finding (71%), and 22% had no detected findings.

Responses to assessed vulnerabilities and/or findings were divided into municipal health service responses (MHS) and non-municipal responses. In total, 17% of children were referred to MHS (Table 4). Of these, 11% also received follow-up in general practice. Non-municipal responses were noted in 81% of all included children, most frequently, the response “follow-up in general practice” (64%) (Table 4). In 47% of all children, this was the only response; 13% of children received no response (Table 4).

There was no statistically significant difference in the distributions of number of pooled vulnerabilities and/or findings between the total population of children and children who received the response “follow-up in general practice” (Wilcoxon rank-sum test, *p* = 0.541). In both populations, children with one vulnerability or finding constituted the largest proportion (47% and 44%, respectively) (Table 5). The children with most vulnerabilities (7–10 vulnerabilities and/or findings) constituted 2% in the total population and 3% of the population receiving the response “follow-up in general practice”.

## 4. Discussion

This study is an attempt to develop our understanding of the concept of child vulnerability from the perspective of general practitioners, by combining qualitative and quantitative methods. Our results offer a deeper understanding of GPs’ perceptions and assessment of child vulnerability during PCHEs. This is aligned with prior Danish studies calling for attention to vulnerable children during PCHEs [21,25,26], as well as international studies highlighting the importance of the GP’s role in caring for vulnerable children and families [2,12,18]. Furthermore, our findings contribute to an understanding of child vulnerability from health care professionals’ perspectives in an international context [5,11]. In the following discussion, we integrate and discuss the main findings from both data sets in three overall topics.

### 4.1. The Complexity of Vulnerability and the Challenges in Recognizing It

Although GPs related vulnerability to psychological, social, and somatic parameters, our results suggest that GPs perceive the term as complex and difficult to define. Several theoretical concepts and models to understand and evaluate child vulnerability in health care exist [16,37,38], including the Danish Strengths and Difficulties Questionnaire [39] which has been suggested as a central element in preventive examinations in Danish general practice [40]. However, some respondents reflected on whether their assessment of vulnerability or challenges was also a product of societal norms and personal beliefs. These reflections have also been found in both Danish and international research, highlighting GPs’ attention towards the challenges involved in applying definitions of “normality” to families and parents, which may reflect their own personal norms [20]. In an international review, Kuruppu et al. [12] similarly underscore GPs’ ability to consider their own norms, values and thresholds in relation to the concept of vulnerability. These reflections resonate with the argument that employing pre-defined categories in the conceptualization of vulnerability risks constraining or narrowing the approach to vulnerable children or potentially undermining independence and agency [6,8].

The challenge of recognizing child vulnerability during PCHEs was also addressed in the interviews. GPs considered the PCHE a relevant space for evaluating child vulnerability, but knowledge of the family and their previous challenges was considered essential in this process. Thus, the significance of the PCHE as the first instance of assessing psychological, social and family-related issues was questioned, as initial findings during PCHEs were often related to the physical development of the child. These findings were also reflected in our quantitative results, where somatic and motor skill challenges were the most common findings. Furthermore, our results underscored that vulnerabilities related to the child were most frequently recorded (“illness and challenges related to the child”), and were almost twice as common as other, more socially informed vulnerabilities. Similarly, interview respondents described how somatic findings with the child were easier to assess during PCHEs, which resonates with findings from previous studies of PCHEs in Danish general practices, where children’s appearance and behavior were also in focus [21]. However, in a recent Danish study, GPs and families expressed openness towards an increased and systematically applied psychosocial focus during PCHEs [25,26], although concerns about time constraints and the sensitive nature of these topics were also noted by the participants. While these concerns were also evident in our study, some GPs considered the somatic focus to be the main purpose of PCHEs. In other Nordic countries with similar preventive services for children, the broader psychosocial focus usually falls within the jurisdiction of the municipality as part of Child Health Services [41,42,43,44]. Current discussions among Danish policymakers about the organizational location and role of PCHEs within general practice [45,46,47] point to the challenges that remain in defining the focus and purpose of PCHEs, and the tensions GPs face when encountering aspects of child vulnerability.

### 4.2. Follow-Up Consultations in General Practice

A second topic was the use of follow-up consultations to build knowledge of the family and their circumstances. In the interviews, GPs described this as essential when in doubt or feeling unsure about how to proceed. This was also evident from the quantitative data, as “follow-up in general practice” was the most frequently applied response by far, and for almost half of all children, this was the only response. What is more, our results showed no statistically significant difference between children receiving the response “follow-up in general practice” and the total population of children, suggesting that “follow-up in general practice” was used equally among groups of children with more or less vulnerabilities and/or findings. Respondents also elaborated how they were dependent on relation to, knowledge of, and collaboration with the parents, and some argued that all challenges should be seen in the light of parental or family resources. Thus, knowledge of the family or parents was considered essential in their assessment of child vulnerability. In prior Danish research, knowledge of the family also has been shown to prompt GPs to include a more family-related assessment during PCHE [21]. This is supported by several international studies, where knowledge of the family has been suggested as a key element in recognizing child vulnerability or challenges within the family [48,49]. However, knowledge of the family has also been suggested to constrain the clinician’s decision to act on signs or suspicions, if they are concerned about jeopardizing the patient’s trust [12,13,49]. This dilemma has also been suggested as central in prior Danish studies, where trust and collaboration with vulnerable families complicated GPs’ responses to suspicion of child abuse [14,50]. Taken together, these findings highlight how some of the inherent challenges of responding to vulnerabilities play a key role in understanding perceptions of child vulnerability.

### 4.3. The GPs’ Collaboration with Social Services

As a third topic, GPs found the cross-sectorial collaboration with social services to be almost non-existent. This contrasted with collaborations with hospitals and private practicing specialists, e.g., pediatricians, psychologists and home health visitors, which were perceived as efficient and well-informed. Quantitative results showed that referrals to municipal health services were made in 17% of cases, but two thirds of municipal health service-referred children (11% of all) also received follow-up in general practice. This suggests that GPs frequently use follow-up consultations in addition to referring to municipal health services, indicating a need to maintain contact with the patient or family after referral. The challenges in collaboration with social services have been highlighted in Danish and international studies [12,14,15,17,18,22,50,51,52], often pointing to a lack of trust in social services [12,18,51]. The absence of adequate feedback and the insufficiency of communication channels, which were key findings in our study, have likewise been identified as central to effective collaboration with social services in previous Nordic [18] and Danish research [14,15,22,35,50]. Further research is vital to develop our understanding of how cross-sectorial trust and collaboration pathways may be improved.

### 4.4. Strengths and Limitations

A limitation of this study is its limited generalizability. Since municipal health services differ between each municipality in Denmark, the data recording chart was adapted to local municipal health services. More knowledge of GPs’ perceptions of child vulnerabilities in other municipalities and on a national basis is vital to gain a more nuanced understanding of the term. Moreover, we did report the local proportion of vulnerable children in the municipality compared to the national proportion, but we did not assess local variance in each general practice, as this information is not accessible. The participating GPs were broadly distributed across years of experience and practice location. However, a more equal distribution among sexes may have provided more nuanced perspectives. Also, all data was pooled due to small numbers, and it was therefore not possible to investigate changes over time from 2020 to 2025, although perceptions of vulnerability could be affected by, e.g., jurisdictional changes in PCHE assessment.

Despite these limitations, we do believe this study represents an important starting point for understanding GPs’ perspectives on, and management of, child vulnerability. Likewise, we find this study’s conceptualization of vulnerability to be an important step towards understanding the multiple dimensions of vulnerability in general practice, albeit highlighting that this represents one version of conceptualizing child vulnerability developed from interviews.

One great strength is the use of different methodologies, underlining both the conceptual challenges of vulnerability and its interpretation and application in clinical practice. The development of the data recording chart from interviews represented an important opportunity to add further validity measures to interview data, although categories were pre-defined based on GPs’ perceptions, which must be included in interpretations. The quantitative data collection has contributed to a better understanding of how children considered in need of extra care are managed, and the interviews have added depth to the complexity of providing this care.

## 5. Conclusions

GPs consider child vulnerability a complex issue and often consider knowledge of the family essential in their evaluation of vulnerability. The PCHE represents one of several clinical encounters in which GPs may assess and respond to vulnerability. Our findings suggest that somatic challenges or issues related to the child are more frequently and easily identified and evaluated during PCHEs, whereas recognition and evaluation of psychosocial factors indicating vulnerability require follow-up consultations. Our findings underline the complexity of understanding, recognizing, and responding to child vulnerability, and underline, once again, how collaboration between general practice and social services is limited and challenging. Our study responds to calls for research into health professionals’ reflections on vulnerability and thus contributes to a broader understanding of child vulnerability in general practice. The uncertainty surrounding this issue, which we found among GPs, could suggest a need for an increased focus on child vulnerability during specialist training. However, our results represent only an initial step, and further research is needed to examine how child vulnerability is perceived, assessed and addressed, using a wider range of experimental and methodological approaches.

## Figures and Tables

**Table 1:** Characteristics of GPs participating in interviews.

Respondents (*n* = 11)	
*% female*	82
Years working as GP (*mean [range], in years*)	10.9 [2–22]
Practice location (*%*)	
Urban	55%
Suburban	18%
Rural	27%

**Table 2:** Assessed vulnerabilities in the family during preventive child health examinations.

Types of Vulnerabilities *	*n* (%)
Family structure	29 (17)
Cultural differences. Language barrier. Different ethnicity	10 (6)
Substance abuse. Criminality	<3 **
Lack of peace and quiet and structure in everyday life	13 (8)
Psychological challenges in parents	30 (18)
Family dynamics. Parent–child relationship. Overly concerned parents	24 (14)
Social challenges and parental ability	11 (6)
Socioeconomical challenges	6 (4)
Somatic disease in parent	11 (6)
Illness + challenges related to the child	57 (33)
**Number of vulnerabilities**	
No vulnerabilities	60 (35)
1 vulnerability	69 (40)
2 vulnerabilities	22 (13)
3–4 vulnerabilities	15 (9)
5–7 vulnerabilities	5 (3)
**Total**	171 (100)

* Some families may display multiple vulnerabilities. ** Too few responses.

**Table 3:** Findings detected in the child during preventive child health examinations.

Types of Findings *	*n* (%)
Psychological and developmental challenges in the child	30 (18)
Social challenges and failure to thrive in the family	15 (9)
Somatic and motor skill challenges in the child	102 (60)
**Number of findings**	
No findings detected	38 (22)
1 finding	122 (71)
2 findings	8 (5)
3 findings	3 (2)
**Total**	171 (100)

* Some children may display multiple findings.

**Table 4:** Responses noted during preventive child health examination when vulnerabilities or findings are detected: municipal health service responses (MHS responses) and non-municipal responses.

	*n* (%)
MHS responses *	29 (17)
MHS responses only	10 (6)
MHS response and follow-up in general practice	19 (11)
Non-municipal responses *	138 (81)
Non-municipal responses only	119 (70)
Follow-up in general practice	109 (64)
Follow-up in general practice only	81 (47)
Child referred to hospital	13 (8)
Child referred to the child and adolescent psychiatry	0 (0)
Parent referred to psychological counseling or similar help	<3 **
Child referred to private pediatrician	5 (3)
Child referred to private physiotherapist	0 (0)
Child referred to private otolaryngologist or ophthalmologist	21 (12)
**No responses noted**	23 (13)
**Total**	171 (100)

* Some children may display multiple responses. ** Too few responses.

**Table 5:** Distribution of number of vulnerabilities and/or findings in total population and in children with “follow-up in general practice” response during preventive child health examinations.

Number of Vulnerabilities and/or Findings	*n* (% of Total Population)	*n* (% of Children with “Follow-Up in General Practice” Response *)
1	80 (47)	48 (44)
2	58 (34)	37 (34)
3–4	21 (12)	14 (13)
5–6	9 (5)	7 (6)
7–10	3 (2)	3 (3)
Total	171 (100)	109 (100)

* Some children may display multiple responses together with follow-up in general practice.

## Data Availability

The data presented in this study are available on request from the corresponding author due to legal and ethical reasons.

## Abbreviations

The following abbreviations are used in this manuscript:

GP	General practitioner
PCHE	Preventive child health examination
MHS	Municipal health services

## Appendix A

### Appendix A.1. Vulnerabilities in the Family

In total, 47 codes of vulnerabilities were merged from the interviews. Codes were firstly reduced to 24 vulnerabilities/subtopics and secondly to 10 topics. One subtopic was excluded as it would be included indirectly in several of the other topics (knowledge of the family). In the category of substance abuse, criminality was added to the topic although this was not mentioned as a risk factor in interviews, since it has been described in the literature as a factor indicating vulnerability, often linked to substance abuse [4,10].

**Table A1:** Vulnerabilities in the family—codes, subtopics and topics.

Code	Subtopic	Topic
Poor social network, socially disadvantaged family background	Poor social network, socially disadvantaged family background	Family structure
Death of a close family member, loss of important resources (e.g., grandparents)		
Problems in the parents’ relationship, parental dynamics, disagreements between parents	Family structure. Siblings. Parents’ relationship, disagreements between parents	
First child		
Sibling, e.g., multiple siblings or ill siblings		
Young parents/young mother	Young parents	
Single parent	Parents divorced. Single parent	
Parents divorced		
Different ethnicity, cultural differences, language barriers (e.g., interpreter needed)	Different ethnicity, cultural differences, language barriers (e.g., interpreter needed)	Cultural differences. Language barrier. Different ethnicity
Substance abuse in parents	Substance abuse	Substance abuse. Criminality
Adversities for the child: lack of peace and quiet and structure in everyday life	Adversities for the child: lack of peace and quiet and structure in everyday life	Lack of peace and quiet and structure in everyday life
Mental illness, stress (in parents)	Mental illness, stress, signs of stress in parents, postpartum depression, complicated delivery	Psychological challenges in parents
Parental resources, signs of stress in parents		
Complicated delivery		
Postnatal depression		
Suboptimal parenting practices, the handling of the child, unusual behavior	Family dynamics. Recognizing the child’s needs. Compassionate. Interacting with each other. Parent–child relationship	Family dynamics. Parent–child relationship. Overly concerned parents
Relationship between parent and child, dynamics within the family, interaction patterns, parental contact		
Parents showing empathy toward the child, reflecting together with the child, emotional understanding/attachment, recognizing the child’s needs, showing care		
Excessive focus on the child’s health. Fear of illness on behalf of the child	Excessive focus on the child’s health. Fear of illness.	
Insecure parents, overly concerned, frequent contact with the general practitioner for minor issues	Overly concerned parents. Insecure. Frequent contact with general practice	
Reduced cognitive ability	Reduced cognitive ability, parental suitability, flattened affect, unengaged	Social challenges and parental ability
Flattened affect, unengaged		
Reduced parental ability		
Laissez fair approach from the parents		
Limited understanding of illness, lack of sense of what is normal regarding the child’s health	Limited understanding of illness	
Residential area	Residential area is socially disadvantaged	
Child appears unwashed, wears clothed with holes and similar signs.	Child appears unwashed, wears clothes with holes in them	
Socially poorly functioning parents	Socially poorly functioning parents	
Parents appear unable to care for themselves, severely overweight, including how they dress and present themselves outwardly	Parents appear unable to care for themselves, severely overweight, including how they dress and present themselves outwardly	
The family has previously missed many appointments	Decline vaccinations. Frequently miss appointments	
Parents decline vaccinations		
Family moves frequently	Family moves frequently	
Unemployment	Socioeconomic conditions, education, unemployment, finances	Socioeconomical challenges
Low level of education		
Socioeconomic conditions, overall resources		
Somatic disease in parent, tapering, for example	Somatic disease in parent	Somatic disease in parent
Medical treatment in parents		
Multiple diseases		
Prolonged sick leave of a parent	Prolonged sick leave of a parent	
Somatic disease in child	Illness, disability, neurodevelopmental disorder in the child. Prematurely born. Ill during the neonatal period	Illness and challenges related to the child
Child with neurodevelopmental disorder		
Child with disability		
Seriously ill child/children		
Prematurely born child, child ill during the neonatal period		
Child has increased illness-related absence from school/daycare		
Family is connected to municipal health services, such as the family group, speech therapy, sensory–motor training through kindergarten, or home health visitors/public health nurse	Knowledge of the family	Excluded (see above)
Family history, knowledge of the parents, vulnerable pregnancy, family support home		

### Appendix A.2. Findings in the Child

In total, 27 codes of findings were merged from the interviews. Codes were firstly reduced to 23 findings/subtopics and secondly to 3 topics. One subtopic was excluded as it would be included indirectly in several of the other topics (daycare reports challenges through the parents).

**Table A2:** Findings in the child—codes, subtopics and topics.

Codes	Subtopic	Topic
Language not age-appropriate	Language not age-appropriate	Psychological and developmental challenges in the child
Stomach pain (no somatic explanation)	Stomach pain (without somatic/physical explanation)	
Social interaction impairments, hyperactive, introvert	Social interaction impairments, hyperactive, introvert	
Tics	Tics	
Signs of neurodevelopmental or mental health difficulties	Signs of neurodevelopmental or mental health difficulties	
Suspicion of attachment-related difficulties	Suspicion of attachment-related difficulties	
Signs of stress in the child, mental well-being, externalizing behavior at home	Signs of stress in the child. Mental well-being. Externalizing behavior	
Sleep disturbance	Sleep disturbance	
Child has difficulties with social relationships, limited social skills	Child has difficulties with social relationships, limited social skills	Social challenges and failure to thrive in the family
Suspicion of abuse	Suspicion/gut feeling of challenges in the family	
A sense that something is not right		
Lack of eye contact and difficulties engaging with the child	Atypical reactions in the child	
Child cries in an unusual way		
Sibling jealousy	Sibling jealousy	
Incontinence, constipation, toilet training, bedwetting, etc.	Incontinence, constipation, toilet training, bedwetting	Somatic and motor skill challenges in the child
Height and weight deviate, overweight, does not gain weight	Overweight. Declining height/weight curves. Does not gain weight	
Eczema, skin problems	Skin problems	
Asthma, asthmatic conditions	Asthmatic conditions	
Ear and hearing problems	Hearing and ear problems	
Vision, eye symptoms, strabismus	Vision and eye symptoms	
Testes present in the scrotum	Testes present in the scrotum	
Structural deformities, curved back, flat feet, etc.	Structural deformities and hypotonic children	
Soft, hypotonic		
Affected gait, ability to stand and walk, toe-walking	Delayed motor development. Sensorimotor challenges	
Delayed motor skill and child has low level of physical activity, sensorimotor challenges		
Heart murmur	Heart murmur	
Daycare reports something is wrong	Daycare reports challenges through the parents	Excluded (see above)

### Appendix A.3. Responses

All non-municipal response topics were mentioned by the respondents, but “private ophthalmologist” was added to the response “private otolaryngologist” since both are private practicing specialists, whom children are often referred to by general practitioners.

Of municipal health service responses, the response “Day care for child with asthma, atopic dermatitis or allergy (Hyldehaven)” was not mentioned by respondents but found on the internet home page for municipal health services [32]. Also, the Family Support was not mentioned by respondents (in the first recording chart “Family counseling in cases of poor well-being or parenting-related problems”), but this response was added since it was available as a municipal health service response and because related codes were covered either by this response or one of the other municipal interventions (e.g., the Family Group). The response “Counseling at early concern for a child’s well-being” was no longer available as a separate health service, but included in other services, e.g., the Family Group. However, a new health service had been created and was included instead (incontinence in children and adolescents). All other municipal health services were mentioned by respondents and available on the internet home page for municipal health services.

**Table A3:** Non-municipal health service responses and municipal health services responses—codes and topics.

Non-Municipal Health Service Response: Code	Topic
Follow-up consultation with practice nurse	Follow-up in general practice
Follow-up consultation for parents without the child	
Follow-up consultation with general practitioner	
Child referred to pediatric unit	Child referred to hospital
Child referred to physiotherapy at the hospital	
Child referred to the child and adolescent psychiatry	Child referred to the child and adolescent psychiatry
Parent referred to psychological counseling or similar help	Parent referred to psychological counseling or similar help
Child referred to private pediatrician	Child referred to private pediatrician
Child referred to private physiotherapist	Child referred to private physiotherapist
Child referred to private otolaryngologist (ears–nose–throat specialist)	Child referred to private otolaryngologist or ophthalmologist
**Municipal health service response: Code**	**Topic**
Day care for child with asthma, atopic dermatitis or allergy (Hyldehaven)	Day care for child with asthma, atopic dermatitis or allergy (Hyldehaven)
The Family Group: special support for children and families	The Family Group * (changed name from” The family group: special support for children and families”)
The Family’s House	The Family’s House * (changed name from “Family House”)
Child psychological counseling/team	The Family Support * (changed name from “Family counseling in cases of poor well-being or parenting-related problems”)
Municipal support person/contact at home/support contact person/home visitor	
Contact to the child’s daycare	Contact to the child’s daycare
Support for overweight in children and adolescents	Support for overweight in children and adolescents * (changed name from “support for overweight in children and adolescents: the Habit Breakers”)
PPR—Pedagogical Psychological Counseling	PPR—Pedagogical Psychological Counseling
Joint consultation regarding child or adolescent	Joint consultation regarding child or adolescent
Public health nurse/home health visitor 0–6 years	Public health nurse 0–6 years
Physical training of children 0–6 years	Physical training of children 0–6 years
Interdisciplinary team (counseling on children 0–6 years)	Incontinence in children and adolescents * (changed from “Counseling at early concern for a child’s well-being”)
Counseling at early concern for a child’s well-being	
Mandatory report	Mandatory report

* The five changes to municipal health service responses from the first data recording chart are marked with *.
